# Hao-Fountain syndrome and genital disorders: report of a new possible association

**DOI:** 10.1186/s13052-022-01367-7

**Published:** 2022-10-22

**Authors:** Nicola Zampieri, Rebecca Pulvirenti, Eleonora Pedrazzoli, Francesco Saverio Camoglio

**Affiliations:** 1 Pediatric Surgery Unit, Woman and Child Hospital, Azienda Ospedaliera Universitaria Integrata, Department of Surgery, Dentistry, Pediatrics and Gynaecology, Pediatric Fertility Lab, University of Verona, Piazzale A. Stefani n.1, 37134 Verona, Italy; 2 Pediatric Surgery Unit, Department of Women’s and Child’s Health, University Hospital of Padova, Padova, Italy; 3 Department of Surgery, Dentistry, Pediatrics and Gynaecology, Anesthesia and Intensive Care Unit, University of Verona, Verona, Italy

**Keywords:** Hao-Fountain, usp7, Tubal torsion, Female

## Abstract

**Background:**

Hao-Fountain syndrome is a neurodevelopmental disorder characterized by global developmental delay, variably impaired intellectual development with significant speech delay and, in some males patients, it has been reported an association with hypogonadism. At present less than 50 cases are reported in literature.

**Case presentation:**

We report a case of this rare syndrome in a young female with isolated tubal torsion; our patients had different hospitalizations without treatment but during the last episode we decide to perform an abdominal surgical explortion. This is the first case in Literature with a new USP7 mutation.

**Conclusions:**

This case opens new perspective in this rare syndrome and a review approach to isolated tubal torsion. These symptoms should be always well checked.

## Background

Hao-Fountain syndrome (HAFOUS) is a neurodevelopmental disorder characterized by global developmental delay, variably impaired intellectual development with significant speech delay, behavioral abnormalities and mild dysmorphic facies. Different clinical aspects are reported and may include, for example, hypotonia, feeding problems, delayed walking, pubertal delay and, in males, it has been reported hypogonadism (undescended testes and micropenis). Less is known about females and gynecological problems [1, 2].

These Authors reported some interesting data: Hao et al. [1] reported 6 unrelated children with variable neurodevelopmental disorders associated with de novo heterozygous microdeletions of chromosome 16p13.2 and 1 patient with a de novo heterozygous truncating variant in the Ubiquitin-specific protease 7 (USP7) gene (602519) on chromosome 16p13.2. All had developmental delay and intellectual disability, and they were diagnosed with autism spectrum disorders. Fountain et al. [2] reported other 11 patients with a similar developmental disorder. Also, in these patients a putative USP7 mutation was identified. They all had developmental delay with variably impaired intellectual development and significant speech delay. Clinical and radiological data about these patients are present into their studies. All these patients had different genetic variants that may have contributed to the phenotype.

Different aspects may be associated with a mutation of the USP7 gene; mutations are either point mutations or gene deletions. Mutations are diagnosed through either whole-exome sequencing or chromosome microarray analysis. The inheritance pattern of the disease caused by USP7 mutations is autosomal dominant, which means that someone who receives a single copy of an abnormal USP7 gene from either parent may develop this disorder. To date most cases are de novo, meaning that the mutation/deletion has been spontaneous and not inherited.

Hao-Fountain Syndrome shares characteristics with many other neurodevelopmental disorders and many patients are initially misdiagnosed. Accurate diagnosis requires microarray or whole exome sequencing.

Whole exome sequencing can provide a diagnosis of Hao-Fountain Syndrome by detecting even very small DNA mutations, of a single letter (point mutations), in the USP7 gene. Sequencing of USP7 alone can bring to the diagnosis, but at present it is difficult to find clinical laboratories offering this specific test. Once the syndrome is diagnosed certain tests are recommended for each patient. These tests include: Measurement of IGF-1 and IGF-BP3 to screen for growth hormone deficiency, a brain MRI (magnetic resonance imaging), a full assessment by a speech pathologist, a full assessment for physical and occupational therapy, formal cognitive and behavioral testing, a sleep apnea test/sleep study, an EEG (electroencephalogram), test to test for abnormal electric activity that could cause/predispose to seizures, a consultation with a gastroenterologist for any reflux, vomiting or chronic constipation/diarrhea issues and an assessment by a pediatric ophthalmologist.

## Case presentation

J.C. (15 years old) is the daughter of consanguineous parents (first degree cousins); she was born at the end of pregnancy. The study was approved by the IRB (Fert Lab 3.0-02-2022). Written informed sonsent was obtained also from the parents for publication of this case report and accompayming images.

One sister died at the age of 3 for cardiomyopathy, a problem from which other relatives would also be affected. Three brothers and one sister are healthy. The stages of psychomotor development were initially regular, but later on a modest delay of language was evidenced (at the age of 3). At the age of 7 she was diagnosed with mild intellectual disability (later re-evaluated as medium) with developmental disturbance of motor function. At the age of 9 she had two episodes of seizures, which did not recur later and the EEG (electroencephalogram) showed mild abnormalities; she has never taken any medication. The brain MRI did not show abnormalities of the brain parenchyma. She had menarche at 12 years with subsequent unregular flow. She has arched eyebrows, horizontal eye rims with bilateral epicanthus, nose with wide root and bulbous tip, regular mouth, microretrognathia, normoimplanted ears with abundant lobe and regular hands and feet. Compared to the previous psychological evaluation, at the age of 13 the patient started to refer greater disesteem with respect to her image; she verbalized an accentuated feeling of inadequacy, asking whether she made a mistake. She also verbalized with a perception of isolation. She currently has difficulties in coordination tests, in the realization of more complex praxis, in the management of a sequence of movements with, for example, difficulties in tying her shoes. She has a hypotonic attitude with lowering of the shoulders. A decreased sensitivity to pain was also referred.

From the genetic point of view, the karyotype analysis was carried out with normal results (46 XX) and the examination for Martin-Bell - X -syndrome excluded the presence of premutation.

Considering the clinical history and the phenotype (based on previous hospitalizations, symptoms and difficulties) the analysis of the exome involved in the HAFOUS development (WES, whole exome sequencing) was made. The analysis was firstly conducted on the patient’s DNA, with the hypothesis of a disease with autosomal recessive transmission based on the consanguinity of the parents. No homozygosity mutations were identified in the patient’s DNA. The re-evaluation of the data allowed to highlight the mutation c.483 + 1G > A in heterozygosity (on a single copy of the gene) in USP7. This mutation has not been identified in the parents, so it must be considered a de novo mutation. During tests, also different variants as 16–9,017,071-C-T (hg19) and ENST00000344836.4:C.383 + 1G > A have been identified. So, our young patient had large de novo heterozygous deletions affecting USP7.

**Fig. 1:**
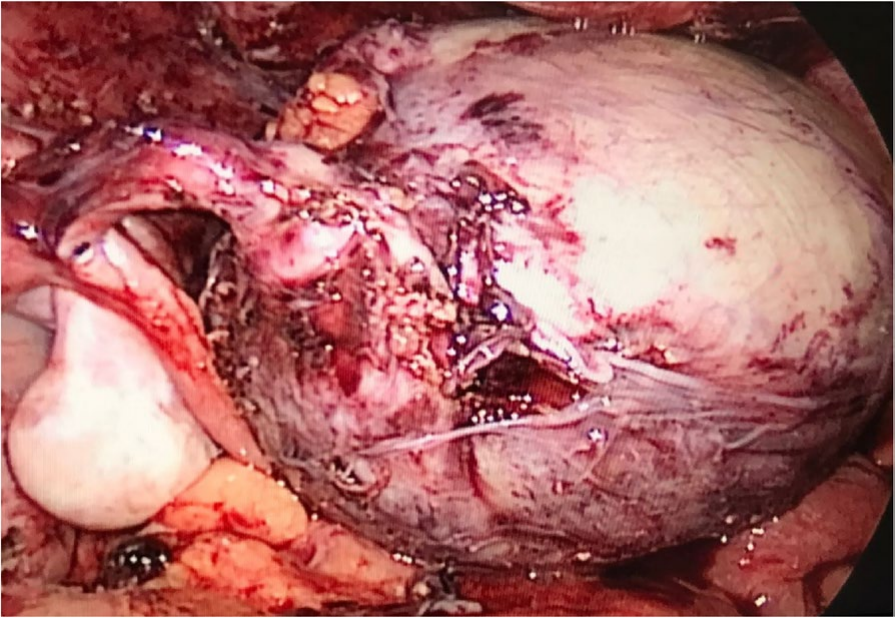
Intraoperative tubal torsion view

On July 2021 she complained abdominal pain, without vomiting or fever, and she was referred to our center. After excluding acute appendicitis, she was evaluated by a gynecologist, who at abdominal ultrasound highlighted a 6-cm wide ovarian cyst with mixed contents. An MRI was performed that confirmed the presence of a cyst attached to the bladder, and showed a normal uterus for age considering that the menstrual cycle was present. Tumoral markers resulted negative (CA125, CA19.9, Alpha-fetoprotein, Beta-HCG, LAD). During laparoscopic surgery a diagnosis of isolated left Fallopian tube torsion (torsion of the distal third) was made (Fig. 1).

The Fallopian tube was necrotic with adhesions to the bladder, the bowel and the iliac vessels. We decided to remove the whole tube. The post-operative course was uneventful and after 2 days of hospitalization the patient was discharged without pain. Histological examination showed a major necrotic transformation of the Fallopian tube without wall or cell’s atypia.

## Discussion and conclusions

Hao-Fountain syndrome is an extremely rare genetic multisystem disorder characterized by different clinical aspects and a heterozygous mutation in the USP7 gene (602519) on chromosome 16p13.2. The disorder is believed to be inherited as an autosomal dominant trait due to USP7 mutations. Ubiquitin-specific protease 7, otherwise known as herpesvirus-associated ubiquitin- specific protease 7 (HAUSP), is a 135 kDa protein and a member of the Deubiquitinating enzymes (DUB) family, which functions in regulating protein stability and localization. It plays a critical role in vital intracellular processes such as epigenetic regulation, cell cycle regulation, cell growth and survival. Of note, its role in stabilizing various tumor suppressors, such as p53, Mdm2, PTEN and Myc, has been well characterized and its upregulation has been associated with many cancers.

USP7 was also observed to play an essential role in growth and development as it was associated with embryonic lethality in USP7 knockout mice [3–10].

It has been reported that USP7 may be implicated in different hormonals balance and in different genital tract disease. For example, some Authors demonstrated that USP7 activity constitutes a novel molecular pathway in modulating the epigenetic state of sex chromosomes during male meiosis and regulating human spermatogonial proliferation via the USP7-mediated p53 signaling pathway. Deubiquitination of Androgen receptor (AR) plays an equally important role in the Androgen Receptors metabolism. Several deubiquitinases have been reported as interacting with the AR and regulating its transcriptional activity; these include USP10, USP26, and USP12. In addition, a recent study demonstrated that USP7 interacts with the AR and facilitates the AR binding to chromatin in prostate cancer cells [2–9].

In males USP7 plays an important role in the expression of a subset of androgen-responsive genes.

In females it plays a role in pregnancy, specifically in the processes involving embryo implantation to the uterine wall. USP7 also regulates ovarian hormones that mediate cell proliferation and function.

It has also been reported that there is a positive correlation between USP7 levels and the estrogen receptor (ER) and the progesterone receptor (PgR) during decidualization. These results suggest that ovarian hormones have effect on USP7 expression and vice versa.

Isolated tubal torsion is a rare emergency and it has been reported to have a right-sided prevalence; this fact has been attributed to the cushioning effect of the sigmoid colon, to the slightly longer right mesosalpinx and to the more frequent exploration of patients with right lower quadrant pain for presumed appendicitis. In some reports, isolated tubal torsion appeared to be more frequent during the premenstrual phase because of the congestion of the mesosalpingeal veins at the time [11, 12].

The most common symptom is ipsilateral lower abdominal pain but a frequent association with chronic pain and eventually nausea and vomiting has been reported; laboratory analysis are usually non-specific [11–15].

The ultrasound evaluation includes normal ovaries with a dilated tube with thickened, echogenic walls and internal debris, representing a twisted tube; other ultrasound findings are a long tubular convoluted cystic structure that tapers toward the uterine cornua, a thin-walled cystic structure with variable septations and mixed internal echoes with visualization of a normal ipsilateral ovary. Color Doppler ultrasonography may demonstrate unilateral absence of blood flow, but the absence of this finding does not necessarily rule out tubal torsion. The value of CT scan and MRI in diagnosing this condition is still controversial [13].

The pediatric literature report, in series or case reports, less than 100 cases; most of these cases were considered secondary to underlying adnexal pathology. Menstrual history, not reported if regular or irregular, was available for 70% of the cases. The youngest patients reported was 4 years old [16].

Salpingectomy has been the standard treatment in case of a clearly necrotic tube, non-reversible ischemia or evidence of secondary tube torsion. Detorsion of the tube, without resection, was first described by Kurzbart; this is typically performed in cases of recent symptoms onset or incomplete torsion, when there is evidence of potentially viable tubal tissue. Promising results were reported following adnexal detorsion but the long term outcomes of this approach are unknown [16, 17].

Conservative treatment has been proposed also by Boukaidi et al. [14], that reported their experience with 6 cases of isolated tubal torsion; their conclusion is to preserve the tube or at least to perform a distal salpingectomy. However, literature has previously suggested that scarred or damaged tube, hydrosalpinx or tubal pathology may result in lower fertility outcomes.

The main question is whether is advisable to perform a conservative treatment or if a salpingectomy is needed.

Especially in this specific case it is essential to remember some important aspects of gynecological cancer and fertility preservation.

More than half of the ovarian cancers occur in the reproductive age group, compromising the reproductive potential; the main factor behind the poor survival rates of ovarian cancer is the stage at presentation and diagnosis.

The origin and the pathogenesis of epithelial ovarian cancer have perplexed investigators for decades. Despite numerous studies that have carefully scrutinized the ovaries for precursor lesions, none have been found. Ovarian cancer is, in fact, the most lethal gynecologic malignancy. Among ovarian cancers it has been reported that, despite well-known and profound differences among the various histologic types, the vast majority of ovarian carcinomas are high-grade serous carcinomas. Ovarian cancer may originate from the ovarian surface epithelium (mesothelium), which invaginates into the underlying stroma resulting in inclusion cysts that eventually undergo malignant transformation. Again, ovarian cancer spreads from the ovary to the pelvis, abdomen and distant sites [18–23].

Recently a new theory has been proposed: the most persuasive data support the view that serous tumors develop from the fimbriated portion of the fallopian tube, endometrioid and clear cell tumors from the endometrial tissue passing through the fallopian tube, resulting in endometriosis, and mucinous and Brenner tumors from transitional-type epithelium located at the tubal-mesothelial junction where the fimbria makes contact with the peritoneum.

Embriologically, the cervix, the endometrium and the fallopian tubes derive from the müllerian ducts, whereas the ovaries develop from the mesodermal epithelium on the urogenital ridge, separate from the müllerian ducts. Therefore, an alternate theory proposes that tumors with a müllerian phenotype (serous, endometrioid and clear cell) are derived from müllerian-type tissue and not mesothelium.

This müllerian-type tissue (columnar epithelium, often ciliated) lines cysts located in paratubal and paraovarian locations that have been referred collectively as the “secondary müllerian system”. According to this theory, ovarian tumors develop from these cysts. As the tumor enlarges, it compresses and eventually obliterates ovarian tissue resulting in an adnexal tumor that appears to have arisen from the ovary. Distal fimbriae end of the fallopian tubes, closer to the ovary, has been considered as primary precursor of high-grade serous carcinoma.

Subsequent studies, in which fallopian tubes were more carefully examined, confirmed that in situ and small, early invasive tubal carcinomas occurred in women with a genetic predisposition for the development of ovarian cancer.

Generally, before a carcinoma acquires the ability to metastasize it must first invade and gain access to blood vessels or lymphatics. Some Authors observed that the fimbria contain a rich angiolymphatic vasculature. Moreover, they are in almost direct contact with the basement membrane of the tubal epithelium and therefore a tubal carcinoma may not need to attain a very large size in order to invade this highly accessible angiolymphatic network.

If it can be unequivocally shown that the serous carcinomas in these women develop almost exclusively in the fimbria, then salpingectomy alone would be sufficient to reduce the risk of ovarian cancer. Accordingly, for women undergoing a hysterectomy for benign uterine disease, removal of only the fallopian tubes with the sparing of the ovaries would improve quality of life and overall survival while still reducing the risk of ovarian carcinoma. Such an approach has important public health implications. This field is important also for the association between impaired fertility potential, Fallopian tube disease and assisted medical procreation [14, 17–19].

Hao-Fountain syndrome comes with different clinical aspects but less is known about the genital and fertility “area”; literature data show the possibility of endocrine complications (pubertal delay, autoimmune diseases), immunological deficit and predisposition to autoimmune diseases and a wide variety of cancers. Moreover, in this case, as suggested by Capra et al. [24], the correct diagnosis allowed us to plan neuropsychiatric, endocrinological, immunological, cardiological, and potential oncological risk follow-up.

The association between USP7 defect and cancer, hypogonadism, infertility and gynecological disorders may add new symptoms to this syndrome. Tubal torsion and ovarian problems in these patients should be excluded in case of abdominal pain. Even if it is well known that menarcheal age is inversely associated with the risk of ovarian cancer, those having adnexal or tubal -ovarian torsion should be followed annually, especially in those case with conervative treatment, in order to avoid future fertility and oncological problems. Miniinvasive surgery seems to be the best option for these young patients as for older female; it is clear that a well oncological expertise help to avoid incomplete resections. This specific mutation may favor and could be associated with some gynecological disorders; our case and this genetic condition offer new inside in the management of tubal torsion [25, 26]; from a clinical perspective the implications of this new paradigm might bring to even more far-reaching consequences.

## Data Availability

The data that support the findings of this study are available from the corresponding author upon reasonable request.

## Abbreviations

HAFOUS: Hao-Fountain syndrome
USP7: Ubiquitin-specific protease 7
IGF: Insuline-like growth factor
MRI: Magnetic resonance imaging
WES: Whole exome sequencing
EEG: Electroencephalogram
ER: Estrogen receptor
PgR: Progesterone receptor
HAUSP: Herpesvirus-associated ubiquitin- specific protease 7
DUB: Deubiquitinating enzymes
